# Identification of RNA-binding protein SNRPA1 for prognosis in prostate cancer

**DOI:** 10.18632/aging.202387

**Published:** 2021-01-15

**Authors:** Penghui Yuan, Le Ling, Xintao Gao, Taotao Sun, Jianping Miao, Xianglin Yuan, Jihong Liu, Zhihua Wang, Bo Liu

**Affiliations:** 1 Department of Urology Tongji Hospital, Tongji Medical College, Huazhong University of Science and Technology, Wuhan 430030, Hubei, China; 2 Department of Geriatrics, Tongji Hospital, Tongji Medical College, Huazhong University of Science and Technology, Wuhan 430030, China; 3 Department of Oncology, Tongji Hospital, Tongji Medical College, Huazhong University of Science and Technology, Wuhan 430030, Hubei, China

**Keywords:** prostate cancer, prognosis, RNA-binding protein, SNRPA1

## Abstract

Prostate cancer is one of the deadliest cancers in men. RNA-binding proteins play a critical role in human cancers; however, whether they have a significant effect on the prognosis of prostate cancer has yet to be elucidated. In the present study, we performed a comprehensive analysis of RNA sequencing and clinical data from the Cancer Genome Atlas dataset and obtained differentially expressed RNA-binding proteins between prostate cancer and benign tissues. We constructed a protein-protein interaction network and Cox regression analyses were conducted to identify prognostic hub RNA-binding proteins. SNRPA1 was associated with the highest risk of poor prognosis and was therefore selected for further analysis. SNRPA1 expression was positively correlated with Gleason score and pathological TNM stage in prostate cancer patients. Furthermore, the expression profile of SNRPA1 was validated using the Oncomine, Human Protein Atlas, and Cancer Cell Line Encyclopedia databases. Meanwhile, the prognostic profile of SNRPA1 was successfully verified in GSE70769. Additionally, the results of molecular experiments revealed the proliferative role of SNRPA1 in prostate cancer cells. In summary, our findings evidenced a relationship between RNA-binding proteins and prostate cancer and indicated the prognostic significance of SNRPA1 in prostate cancer.

## INTRODUCTION

Prostate cancer (PCa) is one of the leading urogenital malignancies worldwide. PCa ranked first in incidence and, following lung cancer, had the second highest mortality rate in American men accounting for approximately 1 in 5 newly diagnosed cases in 2019 [1]. Numerous men with metastatic PCa who receive androgen deprivation therapy develop castration resistance, which is associated with a 5-year mortality rate of over 80% [2, 3]. The pathogenesis of PCa is complex and involves copious genetic aberrations [4]. Therefore, further understanding of the molecular dysfunction and identification of significant biomarkers are critical for early diagnosis and better prognosis in PCa.

RNA-binding proteins (RBPs) mediate the interactions of various RNAs and the formation of ribonucleoproteins to control post-transcriptional regulation of gene expression [5]. Additionally, they play an essential role in regulating the metabolism of RNA, such as splicing, translation, and localization [6]. To date, over 1500 RBPs have been identified in humans [7]. However, the specific biological functions of most RBPs have yet to be fully elucidated. Due to the regulatory roles of RBPs, recent studies have focused on RBP dysfunction in cancer initiation and progression [8, 9]. Reports suggest that Musashi RNA Binding Protein 1 (MSI1) was overexpressed in several tumors of the central nervous system by regulating the Notch signaling pathway [10, 11]. Moreover, the overexpression of RNA-binding motif protein 3 (RBM3) facilitated proliferation and drug resistance via the β-catenin pathway in colon cancer cells [12, 13]; however, it indicated a favorable prognosis in breast cancer [14]. Additionally, eukaryotic translation initiation factor 4E (eIF4E) overexpression led to the development of B cell lymphomas and facilitated lymphomagenesis [15, 16]. Moreover, the inhibition of eIF4E in human tumor xenografts significantly induced apoptosis and suppressed tumor growth [17]. Based on these findings, it is important to conduct systematic research on the role of RBPs in carcinogenesis.

Whether specific RBPs play a critical role in the pathogenesis of PCa has yet to be elucidated. In this study, we performed an integrated analysis of RNA sequencing data from The Cancer Genome Atlas (TCGA) database and identified hub RBP genes related to PCa prognosis. The biological functions and clinical traits of RBPs have been successfully identified and validated in multiple databases and molecular experiments. Therefore, the aim of this study was to enhance the current knowledge by providing novel information regarding PCa-related RBPs and potential biomarkers to better predict the development and prognosis of PCa.

## RESULTS

### Analysis of differentially expressed RBPs in PCa

**Figure 1:**
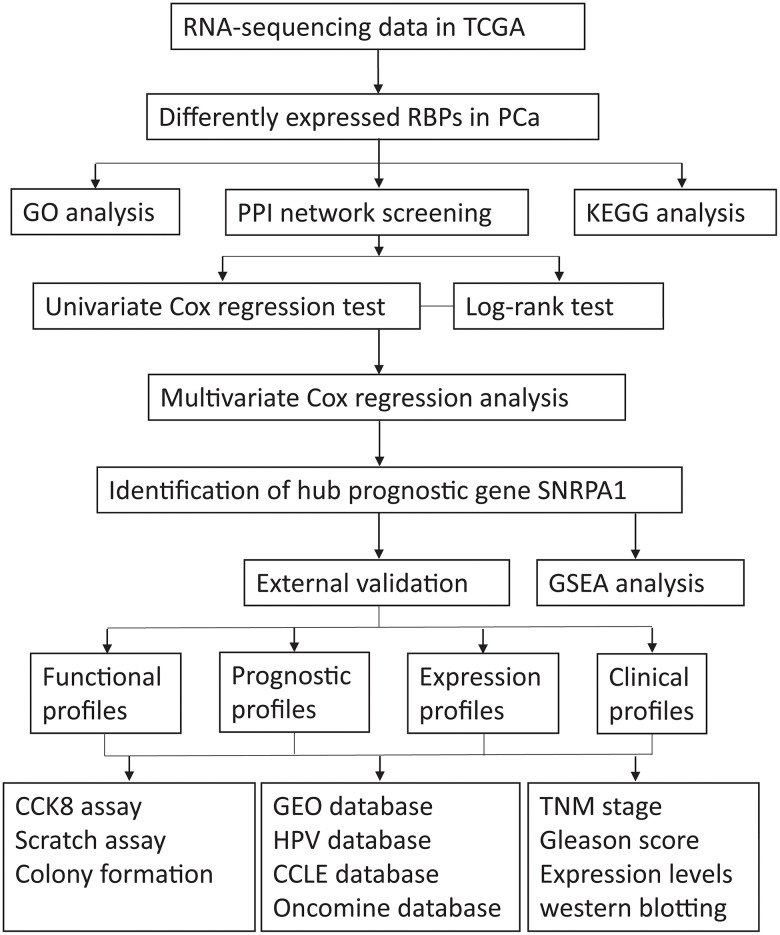
**The flow diagram of the stud.** TCGA: The Cancer Genome Atlas; RBPs: RNA-binding proteins; PCa: Prostate cancer; GO: Gene Ontology; PPI: protein-protein interaction; KEGG: Kyoto Encyclopedia of Genes and Genomes; GSEA: Gene Set Enrichment Analysis; HPA: Human Protein Atlas; CCLE: Cancer Cell Line Encyclopedia; GEO: Gene Expression Omnibus.

**Figure 2:**
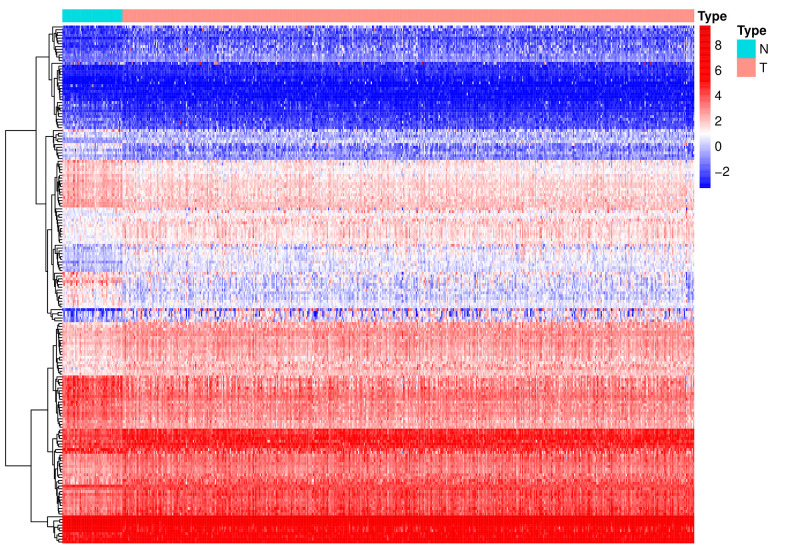
**A heat map of the differentially expressed RBPs between PCa and normal samples.** RBPs: RNA-binding proteins; PCa: Prostate cancer; N: normal issue; T: tumor issue.

A flow diagram of the procedure undertaken in this study is illustrated in Figure 1. After screening for duplicate entries, 495 tumor and 52 normal samples including the mRNA expression profiles of 1472 RBPs were selected from TCGA for further analysis. After processing using the R package, a total of 186 significantly differentially expressed RBPs, including 82 downregulated and 104 upregulated genes, were identified (|log_2_FC| > 0.5 and adjusted p value < 0.0001). A heatmap presenting the expression profiles of RBPs is depicted in Figure 2.

### Identification of biological functions of RBPs

**Figure 3:**
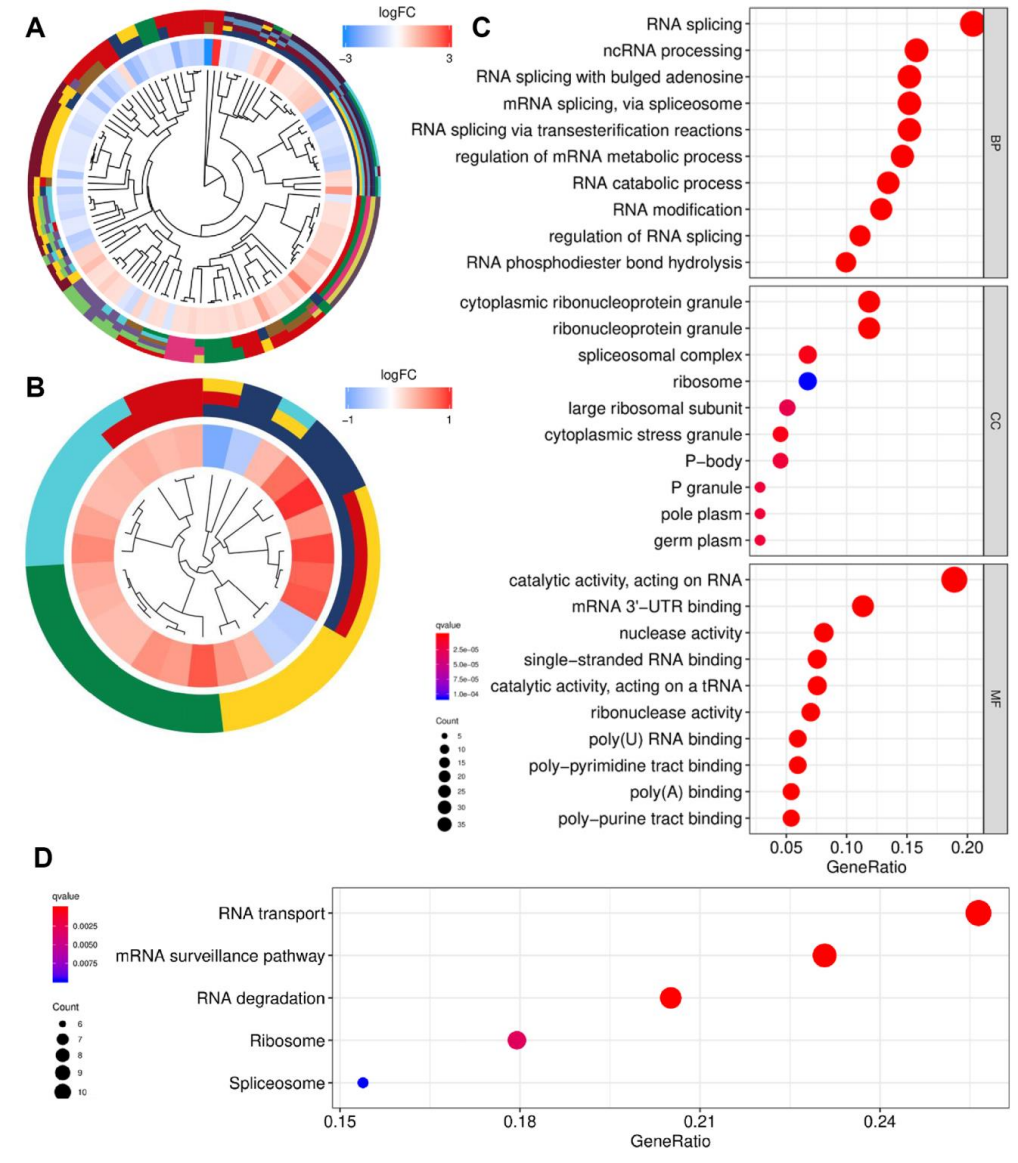
**Functional enrichment analysis of differentially expressed RBPs.** (**A**) GO cluster. (**B**) KEGG cluster. (**C**) GO analysis of representative RBPs including BP, CC and MF. (**D**) KEGG analysis of representative RBPs. For (**A** and **B**), the innermost part shows the hierarchical clustering of the RBPs. The middle part represents the expression profiles of RBPs, in which the color layout from blue to red indicates the expression level of RBPs from down-regulation to up-regulation. And the outermost part represents the GO terms (**A**) and KEGG pathways (**B**) associated with RBPs. The y-axis shows enriched GO terms. RBPs: RNA-binding proteins; GO: Gene Ontology; KEGG: Kyoto Encyclopedia of Genes and Genomes; BP: biological process; CC: cellular component; MF: molecular function.

Gene Ontology (GO) and Kyoto Encyclopedia of Genes and Genomes (KEGG) enrichment analyses were conducted to reveal the biological roles of RBPs. The GO and KEGG profiles are presented in Figure 3A, 3B. RBPs were mainly involved in biological processes including RNA splicing via multiple pathways and mRNA metabolism (Figure 3C). In addition, cellular components significantly associated with RBPs were ribonucleoprotein granules and ribosomes. Moreover, RBPs were significantly enriched in molecular function related to the catalytic and binding activity of RNA. KEGG pathway analysis revealed that RBPs mainly participate in RNA transport, surveillance, degradation, and ribosome activities (Figure 3D).

### PPI network and identification of hub RBPs

**Figure 4:**
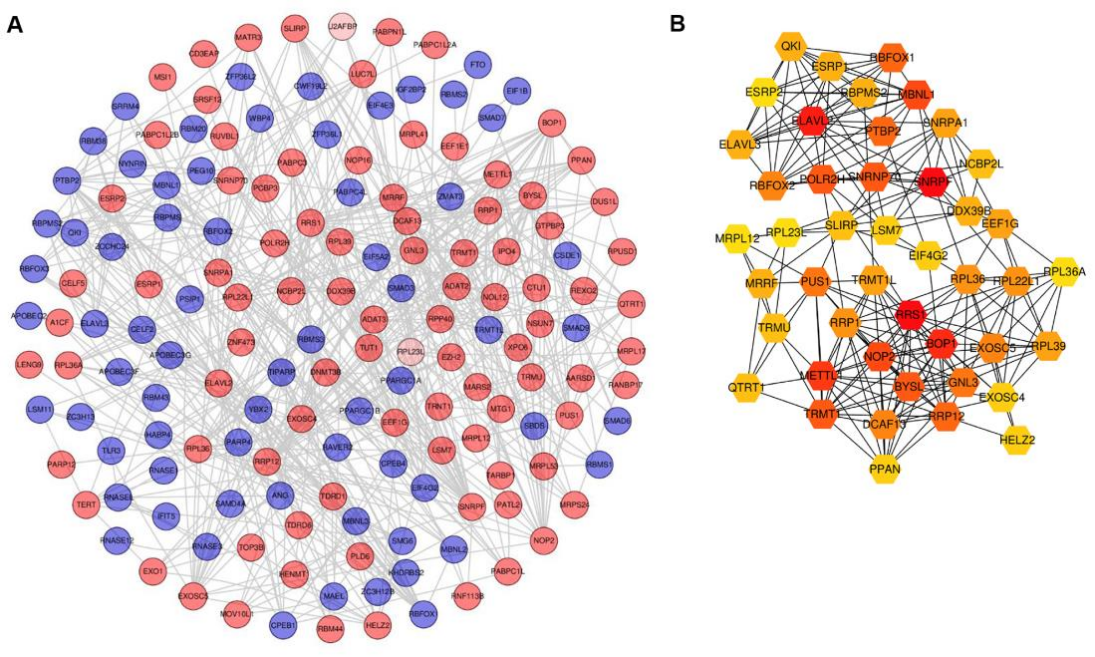
**Screening of differentially expressed RBPs based on PPI network.** (**A**) PPI network of differentially expressed RBPs. (**B**) Gene module based on cytocubba. For A, red circles represent up-regulated RBPs and green circles for down-regulated RBPs. For B, the color layout varying from yellow to red indicates increasing significance of RBPs based on cytocubba. RBPs: RNA-binding proteins; PPI: protein-protein interaction.

To expound RBPs that play a key role during the pathogenesis of PCa, significant RBPs were analyzed using the STRING database. Figure 4A visualizes the protein-protein interaction (PPI) network including 160 nodes and 599 interactions. Significant RBPs were ranked according to degree using Cytoscape plugin cytoHubba. Finally, a gene module comprising 45 hub RBPs was identified (Figure 4B). RRS1, SNRPA1, ELAVL2, and BOP1 were involved in this module.

### Prognostic analysis of hub RBPs

**Figure 5:**
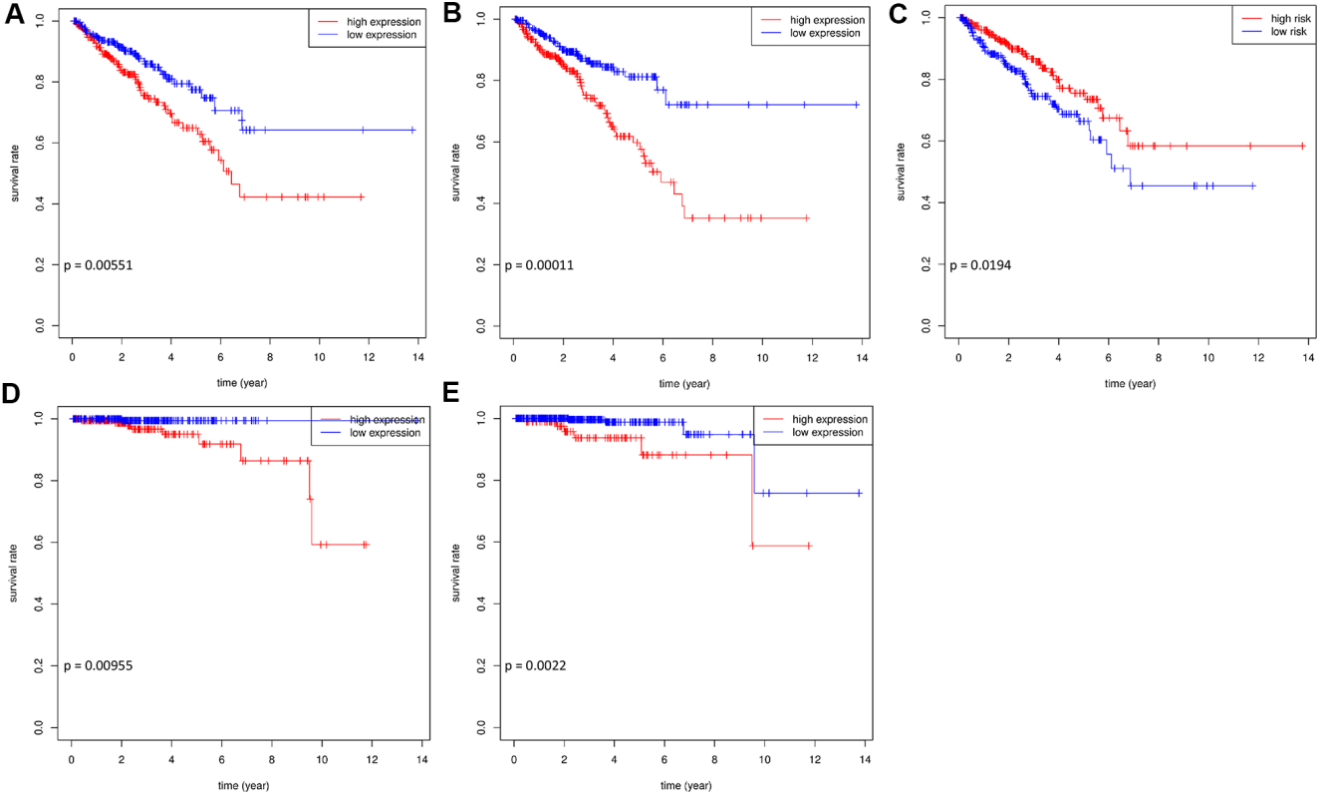
**The survival curves of hub prognostic RBPs.** Prognosis analysis in training and validation datasets. Association between expression of SNRPA1 (**A**), DDX29B (**B**), ESRP2 (**C**) and DFS in TCGA. Association between expression of SNRPA1 (**D**), DDX29B (**E**) and OS in TCGA. RBPs: RNA-binding proteins; DFS: disease-free survival; OS: overall survival; TCGA: The Cancer Genome Atlas.

Of the 45 hub RBPs, 7 candidate genes were related to prognosis with regards to disease-free survival (DFS) in 489 patients, determined by both the log-rank test and univariate Cox regression methods (p value < 0.05). After stepwise multivariate Cox regression analysis, SNRPA1, DDX39B, and ESRP2 remained significant in the model and were regarded as prognosis-related RBPs (p value < 0.05; Supplementary Table 1). The survival curves demonstrated that a higher expression of SNRPA1 and DDX39B and lower expression of ESRP2 were significantly related to worse DFS (p = 0.00551, 0.00011, and 0.0194, respectively; Figure 5A–5C). Furthermore, the expression profiles of SNRPA1 and DDX39B remained significantly associated with overall survival (OS) (p = 0.00955 and 0.0022, respectively; Figure 5D, 5E). This suggests that the abovementioned RBPs are suitable prognostic markers of PCa. SNRPA1 had the highest hazard ratio (HR) value and has not previously been reported in PCa. Based on this, SNRPA1 was selected for further analysis.

### Clinical significance of SNRPA1

**Figure 6:**
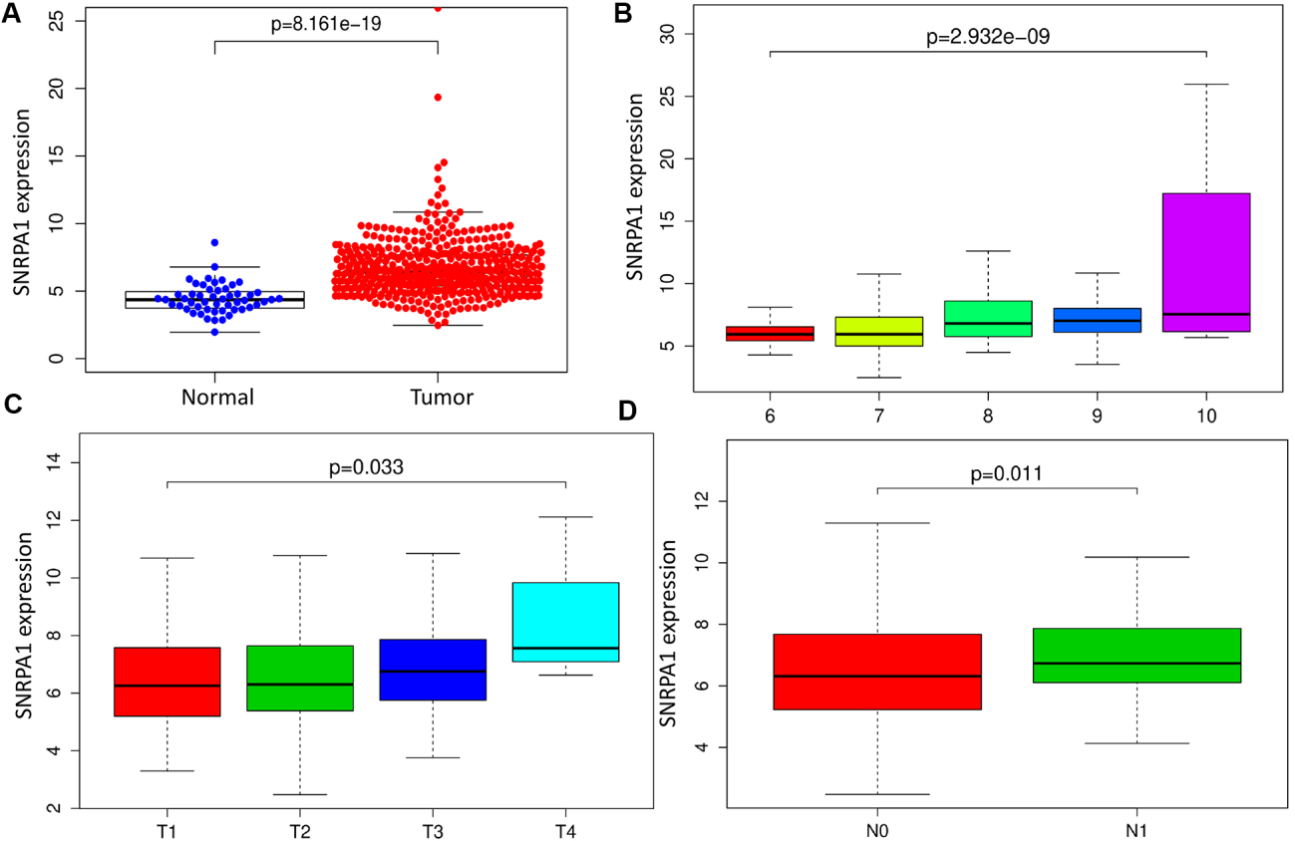
**Clinical significance of SNRPA1.** (**A**) SNRPA1 expression between PCa and normal samples. Association between SNRPA1 expression and Gleason score (**B**), T stage (**C**) and N stage (**D**), respectively. PCa: Prostate cancer.

To reveal the clinical relevance of SNRPA1, we first compared its mRNA expression levels in tumor and normal samples. SNRPA1 was highly expressed in PCa (p < 0.0001; Figure 6A). Additionally, SNRPA1 expression was positively associated with Gleason score (p < 0.0001; Figure 6B). It appeared that SNRPA1 expression increased as the Gleason score increased from 6 to 10. Thereafter, the relationship between pathological TNM stage and SNRPA1 was explored. As depicted in Figure 6C, the T stage demonstrated a similar trend to that of the Gleason score; the higher expression of SNRPA1 corresponded with the advanced T stage (p = 0.033). Finally, samples in the N1 stage had higher expression of SNRPA1 compared with those in the N0 stage (p = 0.011; Figure 6D). Since less than 5 samples exhibited distant metastasis, the correlation between SNRPA1 and M stage was not evaluated. Based on these findings, SNRPA1 evidenced clinicopathological significance in PCa.

### External validation of SNRPA1 across multiple databases

**Figure 7:**
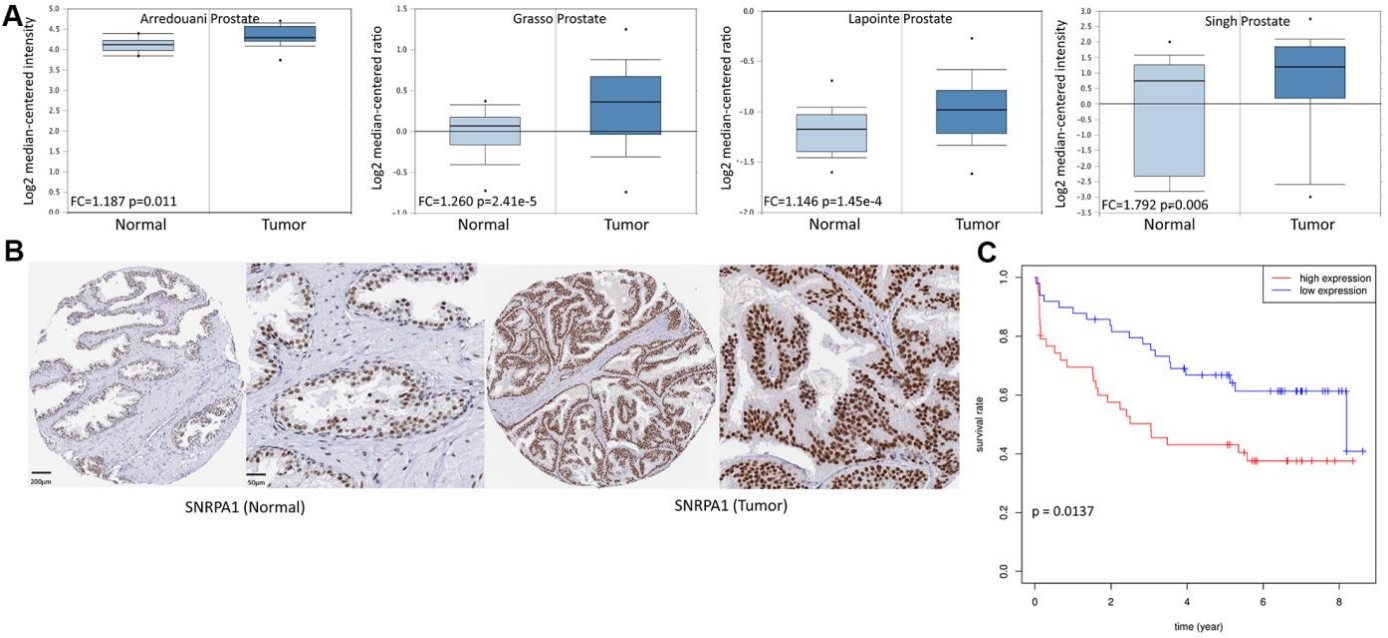
**External validation of SNRPA1 in multiple databases.** (**A**) SNRPA1 expression between PCa and normal samples in four studies of Oncomine. (**B**) The immunohistochemical results of SNRPA1 in HPA. (**C**) The prognostic profile of SNRPA1 with DFS in an independent external cohort GSE70769. PCa: Prostate cancer; HPA: Human Protein Atlas; DFS: disease-free survival; N: normal sample; T: tumor sample.

To further verify the effect of SNRPA1 in PCa, the expression and prognostic profiles of SNRPA1 were evaluated using the Oncomine, Human Protein Atlas (HPA), Gene Expression Omnibus (GEO), and Cancer Cell Line Encyclopedia (CCLE) databases. The Oncomine database contained 4 studies where SNRPA1 expression was significantly higher in PCa tissue compared to that of normal prostate gland tissue (2.2-fold to 2.4-fold increase; p < 0.05; Figure 7A). Similarly, the HPA database demonstrated that SNRPA1 was strongly positive in PCa tissue and moderately positive in normal tissue (antibody HPA045622; Figure 7B). Moreover, the clinical data of 94 patients in GSE70769 were analyzed. Consistent with the prognostic results of DFS and OS from TCGA, increased expression of SNRPA1 in the GSE70769 cohort was significantly associated with worse DFS (p = 0.0137; Figure 7C). Finally, the CCLE database revealed that SNRPA1 was highly expressed in PCa compared to other solid tumor types, except for hematological malignancy, and it was not differentially expressed in various PCa cell lines (Supplementary Figure 1A, 1B).

### External validation of SNRPA1 using clinical specimens and molecular experiments

**Figure 8:**
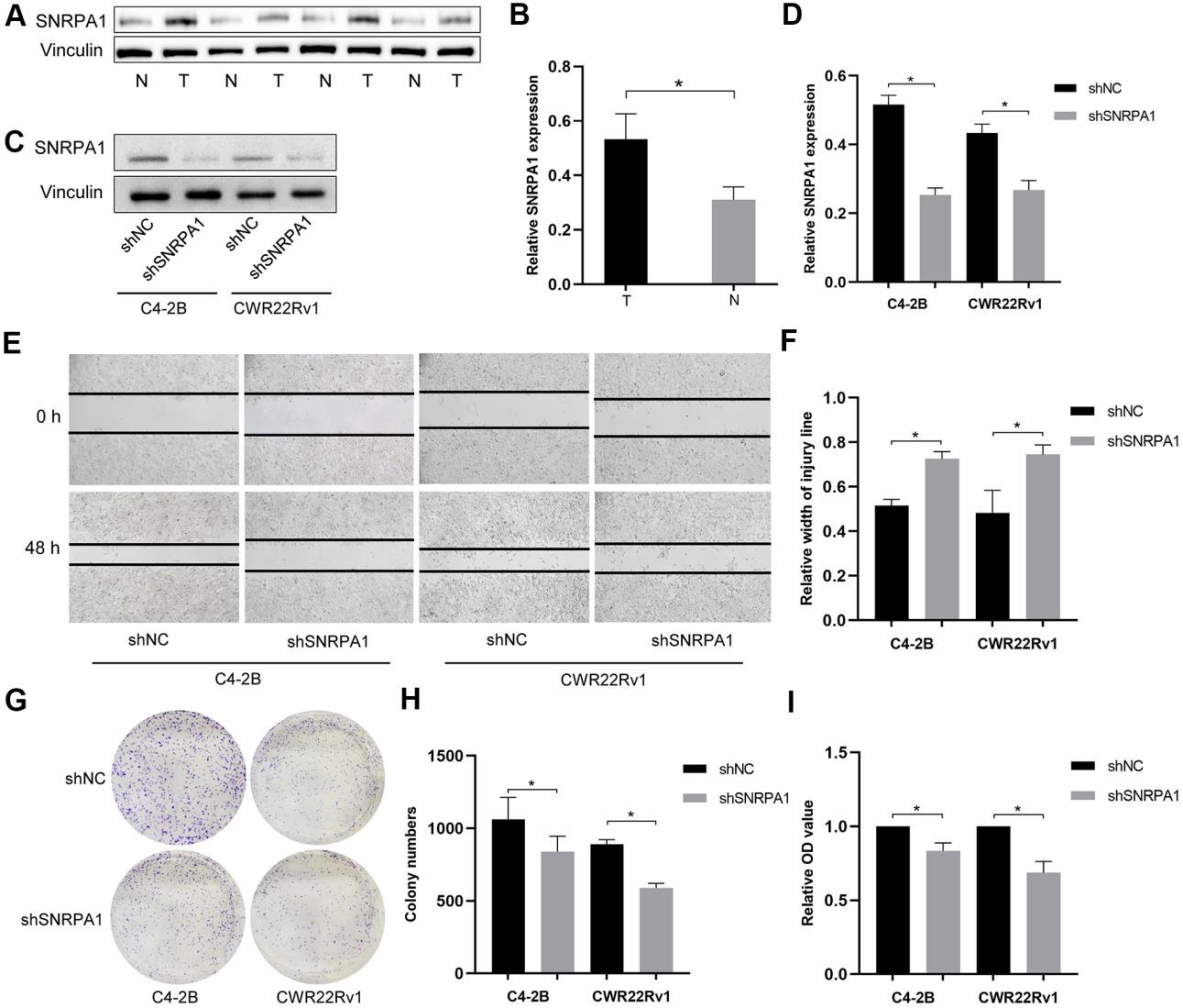
**External validation of SNRPA1 in molecular experiments.** Representative immunoblot (**A**) and quantification (**B**) of SNRPA1 expression between PCa and normal clinical samples. Representative immunoblot (**C**) and quantification (**D**) of SNRPA1 expression after shRNA transfection in CWR22Rv1 and C4-2b cells. Representative results of scratch assay (**E**) and quantification (**F**) of CWR22Rv1 and C4-2b cells. Representative results of colony formation (**G**) and quantification (**H**) of CWR22Rv1 and C4-2b cells. (**I**) The results of cell proliferation using cell counting kit-8 of CWR22Rv1 and C4-2b cells after 48 hours. Data are expressed as means ± SD. *P <0.05. PCa: Prostate cancer; N: normal sample; T: tumor sample; shRNA: short hairpin RNAs; shNC: negative control shRNA; shSNRPA1: shRNA targeting SNRPA1.

In addition to verifying the effects of SNRPA1 across multiple datasets, the expression levels of SNRPA1 in four pairs of PCa and normal samples were detected by western blotting. Consistent with the results from other datasets, the expression of SNRPA1 at protein level was significantly higher in the tumor group compared to that of the normal group (Figure 8A, 8B).

Next, we conducted molecular experiments *in vitro*. After shRNA transfection targeting SNRPA1, the expression levels of SNRPA1 significantly decreased in both CWR22Rv1 and C4–2b cells (Figure 8C, 8D). SNRPA1 inhibition also decreased tumor cell migration and colony formation (Figure 8E–8H). Finally, cell proliferation was significantly inhibited when SNRPA1 was downregulated in CWR22Rv1 and C4–2b cells (Figure 8I). These results reveal SNRPA1 indicates poor prognosis in PCa.

### GSEA of SNRPA1

**Figure 9:**
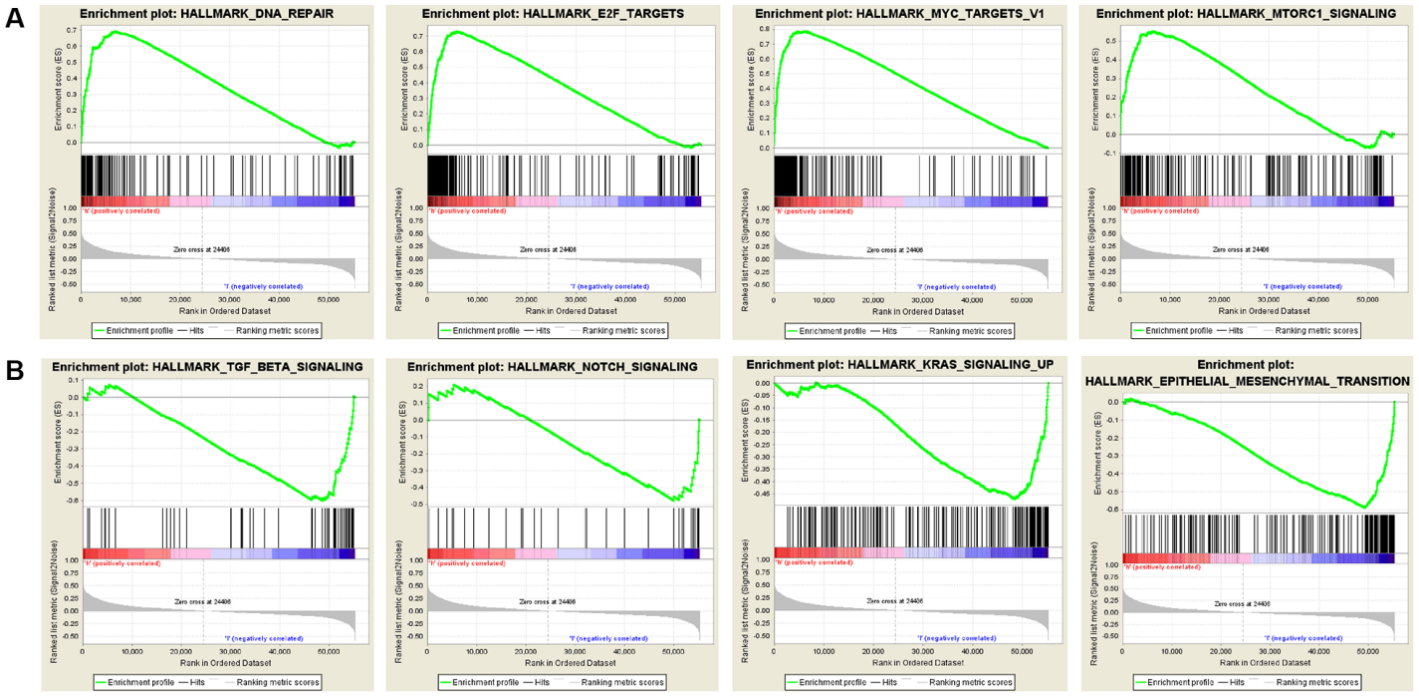
**Significant SNRPA1-related hallmark pathways by GSEA analysis.** (**A**) The most significant pathways associated with up-regulated SNRPA1. (**B**) The most significant pathways associated with down-regulated SNRPA1. GSEA: Gene Set Enrichment Analysis.

Gene Set Enrichment Analysis (GSEA) was conducted using samples with high and low expression levels of SNRPA1 to examine enriched pathways (Figure 9). In the hallmark gene set, the upregulation of SNRPA1 was significantly involved in pathways associated with DNA repair, mTORC1 signaling, MYC, and E2F targets. However, the downregulation of SNRPA1 significantly corresponded to signaling pathways related to TGF-β signaling, Notch signaling, epithelial-mesenchymal transition, and KRAS signaling, which are mainly involved in tumorigenesis.

## DISCUSSION

Globally, PCa is one of the deadliest urogenital tumors in men [18]. Therefore, identifying key genes that can be used as biomarkers is necessary for early diagnosis and prognosis, especially in castration resistance. Many studies have evaluated gene expression profiles in PCa, such as miRNAs, lncRNAs, and autophagy-related genes [19, 20]. However, the systematic evaluation of the role of RBPs in PCa has yet to be reported. Additionally, only a few RBPs have been explored in depth in relation to the pathogenesis of cancer. In this study, we first examined differentially expressed RBPs between PCa and normal samples using RNA sequencing data from TCGA. After analyzing the biological functions of significant RBPs, survival analyses were used to identify prognosis-related RBPs, after which SNRPA1 was selected for further study. The clinical traits of TCGA data demonstrated that SNRPA1 had a positive correlation with Gleason score and TNM stage; higher SNRPA1 expression was related to worse prognosis. Additionally, the expression and prognostic profiles of SNRPA1 were successfully validated by GEO, Oncomine, HPA, and CCLE data. Finally, molecular experiments that downregulated SNRPA1 expression evidenced inhibitory functions in PCa cells. These results provide novel information thereby enhancing our understanding of the role of RBPs in PCa development and prognosis.

Using the threshold of |log_2_FC| > 0.5, we identified differentially expressed RBPs, which were associated with GO terms and KEGG pathways. We evidenced that RNA splicing was the most significant biological process related to RBPs. Changes in mRNA splice patterns play a key role in the pathogenesis of PCa [21]. Gene function could be altered by mRNA isoform switching in PCa through the use of alternative promoters via the androgen receptor (AR). For example, TSC2, a normal tumor suppressor gene in PCa, facilitates cell proliferation under the androgen-driven switch in the mRNA isoform [22]. Additionally, the alternative splicing pattern of the TMPRSS2–ERG fusion gene decreased the skipping of two exons associated with more clinically advanced PCa [23]. Moreover, AR mRNA splicing has an important effect on castration resistance, which could promote PCa cell growth when androgen concentrations are low [24]. AR-v7, a common AR splice variant, increased substantially as patients progressed to castration-resistant prostate cancer [25]. Moreover, the results of cellular component analysis focused on ribonucleoprotein granules, which are involved in biosynthesis. RBPs perform functions mainly by forming ribonucleoprotein complexes with RNA targets as well as transporting, supervising, and degrading RNA [26]. Our KEGG pathway analysis revealed that all of these functions were enriched

Significant RBPs in the PPI network with a medium confidence score were further screened and 45 hub genes were identified. The expression of MBNL1 isoforms lacking exon7 inhibits cell viability and induces DNA damage and could be a negative protein implicated in PCa [27]. Lee et al. [28] reported that GNL3 was associated with low DFS and harbored SNPs related to oncogenesis in PCa. Therefore, GNL3 could be a novel metastasis susceptibility gene in PCa. Liang et al. [29] identified RPL22L1 as a diagnostic and prognostic biomarker in PCa as it promoted PCa cell proliferation and invasion *in vitro*. These hub RBPs were involved in the log-rank test as well as the univariate and multivariate COX regression analyses, and SNRPA1, DDX39B, and ESRP2 were identified. The survival curves demonstrated that all three of these RBPs had significant prognostic profiles with regards to DFS. SNRPA1 and DDX39B remained significantly correlated with OS, although more than 90% of patients in this cohort were still alive at the endpoint. Research has evidenced that ESRP2 is highly expressed in primary PCa and associated with disease progression by androgen-dependent splicing switches [30]. Additionally, DDX39B contributes to the generation of AR-V7 in PCa [31]. Therefore, knockdown of DDX39B could lead to dramatically and selectively downregulated AR-v7 expression. Compared with ESRP2 and DDX39B, SNRPA1 indicated a higher chance of survival based on Cox regression analysis but had not previously been reported in PCa. Thus, we focused on SNRPA1 in PCa for further study.

Firstly, we examined the clinical relevance of SNRPA1. SNRPA1 was significantly associated with Gleason score and pathological TNM stages in PCa patients. Furthermore, it appeared that SNRPA1 expression level was positively correlated with Gleason score. Similarly, higher mRNA expression of SNRPA1 was significantly associated with a more advanced TNM stage. Further, SNRPA1 profiles were validated across multiple databases. Consistent with TCGA data, SNRPA1 was highly expressed in PCa samples based on four studies from Oncomine and HPA. Meanwhile, SNRPA1 had a similar predictive capacity of prognosis as GEO data, and the prognostic profile was well validated. Moreover, the results of clinical specimens by western blotting demonstrated a higher expression level of SNRPA1 in PCa tissue. Furthermore, *in vitro* studies revealed that the inhibition of SNRPA1 could significantly decrease PCa cell migration, proliferation, and colony formation. These results indicate SNRPA1 plays a proliferative role in PCa.

SNRPA1, known as small nuclear ribonucleoprotein polypeptide A, belongs to the spliceosome family and is responsible for processing pre-mRNA into RNA [32]. Additionally, SNRPA1 is one of the key players in the regulation of pluripotency-specific spliceosome assembly [33]. Wu et al. [34] evidenced that the loss of SNRPA1 caused insufficient mRNA splicing and resulted in the failure of spermatocyte differentiation. In cancer research, SNRPA1 bound to the insertion allele of rs386772267 related to pancreatic cancer and influenced gene expression associated with RNA processing and decay [35]. Zeng et al. [36] discovered that SNRPA1 is widely expressed in colorectal cancer cell lines and that downregulation inhibits cell proliferation. Similarly, SNRPA1 was highly expressed in hepatocellular carcinoma and correlated with the clinical stage and OS in hepatocellular carcinoma patients [32]. In our study, we conducted GSEA analysis of SNRPA1 in PCa. The results indicated that a high-risk score was associated with DNA repair, mTORC1 signaling, MYC, and E2F targets, which are also mainly involved in the pathogenesis of PCa [37–39]. Based on these results, we propose that the novel RNA-binding protein SNRPA1 plays an essential role in the pathology and prognosis of PCa.

There are some limitations of this study. Firstly, the study did not explore the specific signaling pathways related to SNRPA1 in PCa, although function enrichment and GSEA analyses were conducted. Furthermore, in-depth molecular experiments need to be conducted to reveal more profound profiles of SNRPA1 as well as clinical data for prognostic analysis in PCa.

In summary, we performed a comprehensive analysis of the role of RBPs in PCa based on RNA sequencing and clinical data. After screening using interaction and survival analysis, SNRPA1 was identified as a significant hub gene related to the prognosis of PCa. Finally, the expression and prognostic profiles were evaluated with clinical traits and well validated across multiple databases and molecular experiments. This is the first study to examine the role of RBPs in PCa. Therefore, our results provide novel information to improve our understanding of PCa development and prognosis.

## MATERIALS AND METHODS

### Data acquisition and processing

Transcriptomic data via RNA sequencing of PCa were downloaded from TCGA database (https://portal.gdc.cancer.gov/), containing 498 tumor and 52 adjacent normal tissues as well as clinical information in May 2020. This database includes the mRNA expression profiles of 1472 RBPs, which were used in this research study [7]. Thereafter, differentially expressed RBP genes were calculated between tumor and normal tissues by “Limma” package [40] in R software (Version 3.6.2). Differentially expressed genes (DEGs) with thresholds of |log_2_FC| > 0.5 and adjusted p value < 0.0001 were selected for further analysis.

### Functional enrichment analysis

To reveal the biological functions of significant RBPs, GO, including molecular function, cellular component, and biological process, and KEGG pathway enrichment analyses were conducted by R package “clusterProfiler” [41] with thresholds of adjusted p value < 0.05 and q value < 0.05. The results were visualized using R package “GOplot” [42].

### Screening of hub RBPs based on PPI network

Significant RBPs were put into the STRING database (http://string-db.org) [43] to expound the interactions between nodes. After disregarding disconnected nodes and screening using an interaction score > 0.4, selected RBPs were further visualized by constructing the protein-protein interaction (PPI) network using Cytoscape software (Version 3.7.1) [44]. RBPs with degrees ≥ 10 were identified as hub RBPs using the cytoHubba plug-in [45].

### Prognostic analysis

To identify the prognostic value of hub RBPs, log-rank tests and univariate Cox regressions were executed for survival analysis with clinical data from TCGA [46]. Hub RBPs with p values < 0.05 (in both methods) were regarded as prognosis-related candidate RBPs and included in the multivariate Cox model using stepwise regression. Finally, prognosis-related RBPs, with p values < 0.05, were identified. Thereafter, Kaplan–Meier survival curves were plotted between survival status and different groups according to the medium value or optimal cut-off value for gene expression levels of prognosis-related RBPs. The primary endpoint was disease-free survival (DFS) and the secondary endpoint was the overall survival (OS). P values < 0.05 were regarded as statistical significance.

### Clinical significance in TCGA

To validate the clinical correlation of prognostic RBPs, their expression profiles were compared between tumor and adjacent normal tissues. Additionally, the relationship between clinical information including Gleason score and TNM stage, and expression levels of prognostic RBPs were analyzed. The Wilcox test and Kruskal-Wallis test were used to make comparisons between two groups or multiple groups, respectively. P values < 0.05 were regarded as statistical significance.

### External validation across multiple databases

To further verify the expression profiles of prognostic RBPs, we first used the Oncomine database (http://www.oncomine.com) [47] to compare the transcriptional expression between PCa and normal prostate gland tissues using the Student’s t-test. Thereafter, immunohistochemical data were obtained from the HPA database (https://www.proteinatlas.org/) [48] to compare the levels of protein encoded by genes. Immunohistochemistry results of tumor and normal tissues were detected by the same antibody. Another dataset, GSE70769 [49], including the clinical data of 94 patients, obtained from the GEO database (https://www.ncbi.nlm.nih.gov/geo) was used to validate the predictive capability of prognostic RBPs with DFS as the endpoint. Finally, the CCLE database (http://www.broadinstitute.org/ccle/home) [50] was used to reveal the expression of prognosis-related RBPs in multiple solid tumors and PCa cell lines.

### GSEA analysis

To explore the potential signaling pathways underlying the gene signature between different expression levels of prognostic RBPs, we conducted GSEA with the hallmark (“hallmark.all.symbols.gmt”) gene sets collection based on TCGA [51]. The nominal (NOM) p values < 0.05 and false discovery rate (FDR) q values < 0.25 were regarded as statistical significance.

### Identification of SNRPA1 in molecular experiments

The PCa cell lines CWR22Rv1 and C4–2b were cultured in RPMI1640 medium supplemented with 10% fetal bovine serum. Short hairpin RNAs (shRNAs), targeting SNRPA1 or the shRNA negative control, were transfected into CWR22Rv1 and C4–2b cells. Protein samples were extracted from patients with PCa and shRNA-transfected cells. After electrophoresis and incubation with primary antibodies against SNRPA1 (ab128937; Abcam,) and vinculin (#13901, Cell Signaling Technology) as well as secondary antibodies, the expression levels of target proteins were analyzed with an ECL kit and exposure system (Bio-Rad Laboratories).

To reveal the role of SNRPA1 in PCa cells, a colony formation assay was conducted with CWR22Rv1 and C4–2b cells in 6-well plates. After 2 weeks, cell colonies were fixed with 4% paraformaldehyde, stained with 0.2% crystal violet, imaged using microscopy, and counted using ImageJ software. A scratch assay was performed by scratching straight lines with a 200-μm pipette tip into the monolayer of CWR22Rv1 and C4-2b cells, cultured in 6-well plates. After 48 hours, the cells were imaged using microscopy and the scratch widths were measured to determine the migration and invasion of cells. Finally, cell proliferation was measured by a cell counting kit-8 (C0037, Beyotime) according to the manufacturer's instructions, and assessed by measuring absorbance at 450 nm.

Data analysis was conducted using ImageJ software (1.50i) and Statistical Package for Social Science (SPSS 23.0). P values < 0.05 were regarded as statistically significant. Results were visualized as figures using GraphPad Prism 8.0. This study was approved by the Ethics Committee of Tongji Medical College, Huazhong University of Science and Technology.

## Supplementary Material

Supplementary Figure 1

Supplementary Table 1

## References

[1] Siegel RL, Miller KD, Jemal A. Cancer statistics, 2019. CA Cancer J Clin. 2019; 69:7–34. 10.3322/caac.21551 30620402

[2] Grossman DC, Curry SJ, Owens DK, Bibbins-Domingo K, Caughey AB, Davidson KW, Doubeni CA, Ebell M, Epling JW Jr, Kemper AR, Krist AH, Kubik M, Landefeld CS, et al, and US Preventive Services Task Force. Screening for prostate cancer: US preventive services task force recommendation statement. JAMA. 2018; 319:1901–13. 10.1001/jama.2018.3710 29801017

[3] Moreira DM, Howard LE, Sourbeer KN, Amarasekara HS, Chow LC, Cockrell DC, Pratson CL, Hanyok BT, Aronson WJ, Kane CJ, Terris MK, Amling CL, Cooperberg MR, Freedland SJ. Predicting time from metastasis to overall survival in castration-resistant prostate cancer: results from SEARCH. Clin Genitourin Cancer. 2017; 15:60–66.e2. 10.1016/j.clgc.2016.08.018 27692812 PMC5536956

[4] Gandhi J, Afridi A, Vatsia S, Joshi G, Joshi G, Kaplan SA, Smith NL, Khan SA. The molecular biology of prostate cancer: current understanding and clinical implications. Prostate Cancer Prostatic Dis. 2018; 21:22–36. 10.1038/s41391-017-0023-8 29282359

[5] Wang ZL, Li B, Luo YX, Lin Q, Liu SR, Zhang XQ, Zhou H, Yang JH, Qu LH. Comprehensive genomic characterization of RNA-binding proteins across human cancers. Cell Rep. 2018; 22:286–98. 10.1016/j.celrep.2017.12.035 29298429

[6] Kim MY, Hur J, Jeong S. Emerging roles of RNA and RNA-binding protein network in cancer cells. BMB Rep. 2009; 42:125–30. 10.5483/bmbrep.2009.42.3.125 19335997

[7] Gerstberger S, Hafner M, Tuschl T. A census of human RNA-binding proteins. Nat Rev Genet. 2014; 15:829–45. 10.1038/nrg3813 25365966 PMC11148870

[8] Lukong KE, Chang KW, Khandjian EW, Richard S. RNA-binding proteins in human genetic disease. Trends Genet. 2008; 24:416–25. 10.1016/j.tig.2008.05.004 18597886

[9] Pereira B, Billaud M, Almeida R. RNA-binding proteins in cancer: old players and new actors. Trends Cancer. 2017; 3:506–28. 10.1016/j.trecan.2017.05.003 28718405

[10] Kawahara H, Imai T, Imataka H, Tsujimoto M, Matsumoto K, Okano H. Neural RNA-binding protein Musashi1 inhibits translation initiation by competing with eIF4G for PABP. J Cell Biol. 2008; 181:639–53. 10.1083/jcb.200708004 18490513 PMC2386104

[11] Okano H, Kawahara H, Toriya M, Nakao K, Shibata S, Imai T. Function of RNA-binding protein Musashi-1 in stem cells. Exp Cell Res. 2005; 306:349–56. 10.1016/j.yexcr.2005.02.021 15925591

[12] Chatterji P, Rustgi AK. RNA binding proteins in intestinal epithelial biology and colorectal cancer. Trends Mol Med. 2018; 24:490–506. 10.1016/j.molmed.2018.03.008 29627433 PMC5927824

[13] Venugopal A, Subramaniam D, Balmaceda J, Roy B, Dixon DA, Umar S, Weir SJ, Anant S. RNA binding protein RBM3 increases β-catenin signaling to increase stem cell characteristics in colorectal cancer cells. Mol Carcinog. 2016; 55:1503–16. 10.1002/mc.22404 26331352 PMC5276710

[14] Jögi A, Brennan DJ, Rydén L, Magnusson K, Fernö M, Stål O, Borgquist S, Uhlen M, Landberg G, Påhlman S, Pontén F, Jirström K. Nuclear expression of the RNA-binding protein RBM3 is associated with an improved clinical outcome in breast cancer. Mod Pathol. 2009; 22:1564–74. 10.1038/modpathol.2009.124 19734850

[15] Ruggero D, Montanaro L, Ma L, Xu W, Londei P, Cordon-Cardo C, Pandolfi PP. The translation factor eIF-4E promotes tumor formation and cooperates with c-Myc in lymphomagenesis. Nat Med. 2004; 10:484–86. 10.1038/nm1042 15098029

[16] Wendel HG, De Stanchina E, Fridman JS, Malina A, Ray S, Kogan S, Cordon-Cardo C, Pelletier J, Lowe SW. Survival signalling by Akt and eIF4E in oncogenesis and cancer therapy. Nature. 2004; 428:332–37. 10.1038/nature02369 15029198

[17] Avdulov S, Li S, Michalek V, Burrichter D, Peterson M, Perlman DM, Manivel JC, Sonenberg N, Yee D, Bitterman PB, Polunovsky VA. Activation of translation complex eIF4F is essential for the genesis and maintenance of the malignant phenotype in human mammary epithelial cells. Cancer Cell. 2004; 5:553–63. 10.1016/j.ccr.2004.05.024 15193258

[18] Bray F, Ferlay J, Soerjomataram I, Siegel RL, Torre LA, Jemal A. Global cancer statistics 2018: GLOBOCAN estimates of incidence and mortality worldwide for 36 cancers in 185 countries. CA Cancer J Clin. 2018; 68:394–424. 10.3322/caac.21492 30207593

[19] Hu D, Jiang L, Luo S, Zhao X, Hu H, Zhao G, Tang W. Development of an autophagy-related gene expression signature for prognosis prediction in prostate cancer patients. J Transl Med. 2020; 18:160. 10.1186/s12967-020-02323-x 32264916 PMC7137440

[20] Xiaoli Z, Yawei W, Lianna L, Haifeng L, Hui Z. Screening of target genes and regulatory function of miRNAs as prognostic indicators for prostate cancer. Med Sci Monit. 2015; 21:3748–59. 10.12659/msm.894670 26628405 PMC4671457

[21] Munkley J, Livermore K, Rajan P, Elliott DJ. RNA splicing and splicing regulator changes in prostate cancer pathology. Hum Genet. 2017; 136:1143–54. 10.1007/s00439-017-1792-9 28382513 PMC5602090

[22] Munkley J, Rajan P, Lafferty NP, Dalgliesh C, Jackson RM, Robson CN, Leung HY, Elliott DJ. A novel androgen-regulated isoform of the TSC2 tumour suppressor gene increases cell proliferation. Oncotarget. 2014; 5:131–39. 10.18632/oncotarget.1405 24318044 PMC3960195

[23] Hagen RM, Adamo P, Karamat S, Oxley J, Aning JJ, Gillatt D, Persad R, Ladomery MR, Rhodes A. Quantitative analysis of ERG expression and its splice isoforms in formalin-fixed, paraffin-embedded prostate cancer samples: association with seminal vesicle invasion and biochemical recurrence. Am J Clin Pathol. 2014; 142:533–40. 10.1309/AJCPH88QHXARISUP 25239421

[24] Lu C, Luo J. Decoding the androgen receptor splice variants. Transl Androl Urol. 2013; 2:178–86. 10.3978/j.issn.2223-4683.2013.09.08 25356377 PMC4209743

[25] Cato L, de Tribolet-Hardy J, Lee I, Rottenberg JT, Coleman I, Melchers D, Houtman R, Xiao T, Li W, Uo T, Sun S, Kuznik NC, Göppert B, et al. ARv7 represses tumor-suppressor genes in castration-resistant prostate cancer. Cancer Cell. 2019; 35:401–13.e6. 10.1016/j.ccell.2019.01.008 30773341 PMC7246081

[26] Mitchell SF, Parker R. Principles and properties of eukaryotic mRNPs. Mol Cell. 2014; 54:547–58. 10.1016/j.molcel.2014.04.033 24856220

[27] Tabaglio T, Low DH, Teo WK, Goy PA, Cywoniuk P, Wollmann H, Ho J, Tan D, Aw J, Pavesi A, Sobczak K, Wee DK, Guccione E. MBNL1 alternative splicing isoforms play opposing roles in cancer. Life Sci Alliance. 2018; 1:e201800157. 10.26508/lsa.201800157 30456384 PMC6238595

[28] Lee M, Williams KA, Hu Y, Andreas J, Patel SJ, Zhang S, Crawford NP. GNL3 and SKA3 are novel prostate cancer metastasis susceptibility genes. Clin Exp Metastasis. 2015; 32:769–82. 10.1007/s10585-015-9745-y 26429724

[29] Liang Z, Mou Q, Pan Z, Zhang Q, Gao G, Cao Y, Gao Z, Pan Z, Feng W. Identification of candidate diagnostic and prognostic biomarkers for human prostate cancer: RPL22L1 and RPS21. Med Oncol. 2019; 36:56. 10.1007/s12032-019-1283-z 31089825

[30] Munkley J, Li L, Krishnan SR, Hysenaj G, Scott E, Dalgliesh C, Oo HZ, Maia TM, Cheung K, Ehrmann I, Livermore KE, Zielinska H, Thompson O, et al. Androgen-regulated transcription of ESRP2 drives alternative splicing patterns in prostate cancer. Elife. 2019; 8:e47678. 10.7554/eLife.47678 31478829 PMC6788855

[31] Nakata D, Nakao S, Nakayama K, Araki S, Nakayama Y, Aparicio S, Hara T, Nakanishi A. The RNA helicase DDX39B and its paralog DDX39A regulate androgen receptor splice variant AR-V7 generation. Biochem Biophys Res Commun. 2017; 483:271–76. 10.1016/j.bbrc.2016.12.153 28025139

[32] Feng J, Guo J, Zhao P, Shen J, Chai B, Wang J. mTOR up-regulation of SNRPA1 contributes to hepatocellular carcinoma development. Biosci Rep. 2020; 40:BSR20193815. 10.1042/BSR20193815 32420585 PMC7295620

[33] Kim YD, Lee J, Kim HS, Lee MO, Son MY, Yoo CH, Choi JK, Lee SC, Cho YS. The unique spliceosome signature of human pluripotent stem cells is mediated by SNRPA1, SNRPD1, and PNN. Stem Cell Res. 2017; 22:43–53. 10.1016/j.scr.2017.05.010 28595116

[34] Wu H, Sun L, Wen Y, Liu Y, Yu J, Mao F, Wang Y, Tong C, Guo X, Hu Z, Sha J, Liu M, Xia L. Major spliceosome defects cause male infertility and are associated with nonobstructive azoospermia in humans. Proc Natl Acad Sci USA. 2016; 113:4134–39. 10.1073/pnas.1513682113 27035939 PMC4839444

[35] Hoskins JW, Ibrahim A, Emmanuel MA, Manmiller SM, Wu Y, O’Neill M, Jia J, Collins I, Zhang M, Thomas JV, Rost LM, Das S, Parikh H, et al. Functional characterization of a chr13q22.1 pancreatic cancer risk locus reveals long-range interaction and allele-specific effects on DIS3 expression. Hum Mol Genet. 2016; 25:4726–38. 10.1093/hmg/ddw300 28172817 PMC5815622

[36] Zeng Q, Lei F, Chang Y, Gao Z, Wang Y, Gao Q, Niu P, Li Q. An oncogenic gene, SNRPA1, regulates PIK3R1, VEGFC, MKI67, CDK1 and other genes in colorectal cancer. Biomed Pharmacother. 2019; 117:109076. 10.1016/j.biopha.2019.109076 31203132

[37] Dardenne E, Beltran H, Benelli M, Gayvert K, Berger A, Puca L, Cyrta J, Sboner A, Noorzad Z, MacDonald T, Cheung C, Yuen KS, Gao D, et al. N-myc induces an EZH2-mediated transcriptional program driving neuroendocrine prostate cancer. Cancer Cell. 2016; 30:563–77. 10.1016/j.ccell.2016.09.005 27728805 PMC5540451

[38] Han D, Chen S, Han W, Gao S, Owiredu JN, Li M, Balk SP, He HH, Cai C. ZBTB7A mediates the transcriptional repression activity of the androgen receptor in prostate cancer. Cancer Res. 2019; 79:5260–71. 10.1158/0008-5472.CAN-19-0815 31444154 PMC6801099

[39] Zabala-Letona A, Arruabarrena-Aristorena A, Martín-Martín N, Fernandez-Ruiz S, Sutherland JD, Clasquin M, Tomas-Cortazar J, Jimenez J, Torres I, Quang P, Ximenez-Embun P, Bago R, Ugalde-Olano A, et al. mTORC1-dependent AMD1 regulation sustains polyamine metabolism in prostate cancer. Nature. 2017; 547:109–113. 10.1038/nature22964 28658205 PMC5505479

[40] Ritchie ME, Phipson B, Wu D, Hu Y, Law CW, Shi W, Smyth GK. Limma powers differential expression analyses for RNA-sequencing and microarray studies. Nucleic Acids Res. 2015; 43:e47. 10.1093/nar/gkv007 25605792 PMC4402510

[41] Yu G, Wang LG, Han Y, He QY. clusterProfiler: an R package for comparing biological themes among gene clusters. OMICS. 2012; 16:284–87. 10.1089/omi.2011.0118 22455463 PMC3339379

[42] Walter W, Sánchez-Cabo F, Ricote M. GOplot: an R package for visually combining expression data with functional analysis. Bioinformatics. 2015; 31:2912–14. 10.1093/bioinformatics/btv300 25964631

[43] Szklarczyk D, Morris JH, Cook H, Kuhn M, Wyder S, Simonovic M, Santos A, Doncheva NT, Roth A, Bork P, Jensen LJ, von Mering C. The STRING database in 2017: quality-controlled protein-protein association networks, made broadly accessible. Nucleic Acids Res. 2017; 45:D362–68. 10.1093/nar/gkw937 27924014 PMC5210637

[44] Shannon P, Markiel A, Ozier O, Baliga NS, Wang JT, Ramage D, Amin N, Schwikowski B, Ideker T. Cytoscape: a software environment for integrated models of biomolecular interaction networks. Genome Res. 2003; 13:2498–504. 10.1101/gr.1239303 14597658 PMC403769

[45] Yang JF, Shi SN, Xu WH, Qiu YH, Zheng JZ, Yu K, Song XY, Li F, Wang Y, Wang R, Qu YY, Zhang HL, Zhou XQ. Screening, identification and validation of CCND1 and PECAM1/CD31 for predicting prognosis in renal cell carcinoma patients. Aging (Albany NY). 2019; 11:12057–79. 10.18632/aging.102540 31850854 PMC6949065

[46] Liu F, Liao Z, Song J, Yuan C, Liu Y, Zhang H, Pan Y, Zhang Z, Zhang B. Genome-wide screening diagnostic biomarkers and the construction of prognostic model of hepatocellular carcinoma. J Cell Biochem. 2020; 121:2582–94. 10.1002/jcb.29480 31692036

[47] Rhodes DR, Yu J, Shanker K, Deshpande N, Varambally R, Ghosh D, Barrette T, Pandey A, Chinnaiyan AM. ONCOMINE: a cancer microarray database and integrated data-mining platform. Neoplasia. 2004; 6:1–6. 10.1016/s1476-5586(04)80047-2 15068665 PMC1635162

[48] Asplund A, Edqvist PH, Schwenk JM, Pontén F. Antibodies for profiling the human proteome-The Human Protein Atlas as a resource for cancer research. Proteomics. 2012; 12:2067–77. 10.1002/pmic.201100504 22623277

[49] Ross-Adams H, Lamb AD, Dunning MJ, Halim S, Lindberg J, Massie CM, Egevad LA, Russell R, Ramos-Montoya A, Vowler SL, Sharma NL, Kay J, Whitaker H, et al, and CamCaP Study Group. Integration of copy number and transcriptomics provides risk stratification in prostate cancer: a discovery and validation cohort study. EBioMedicine. 2015; 2:1133–44. 10.1016/j.ebiom.2015.07.017 26501111 PMC4588396

[50] Barretina J, Caponigro G, Stransky N, Venkatesan K, Margolin AA, Kim S, Wilson CJ, Lehár J, Kryukov GV, Sonkin D, Reddy A, Liu M, Murray L, et al. The cancer cell line encyclopedia enables predictive modelling of anticancer drug sensitivity. Nature. 2012; 483:603–07. 10.1038/nature11003 22460905 PMC3320027

[51] Subramanian A, Tamayo P, Mootha VK, Mukherjee S, Ebert BL, Gillette MA, Paulovich A, Pomeroy SL, Golub TR, Lander ES, Mesirov JP. Gene set enrichment analysis: a knowledge-based approach for interpreting genome-wide expression profiles. Proc Natl Acad Sci USA. 2005; 102:15545–50. 10.1073/pnas.0506580102 16199517 PMC1239896

