# Supplementary material for: Identification of RNA-binding protein SNRPA1 for prognosis in prostate cancer
Source: Aging (Albany NY). 2021 Jan 15;13(2):2895–911. doi: 10.18632/aging.202387 (PMC7880319; doi:10.18632/aging.202387)
Supplement: Supplementary Figure 1 [file aging-13-202387-s001.pdf]

SUPPLEMENTARY FIGURE

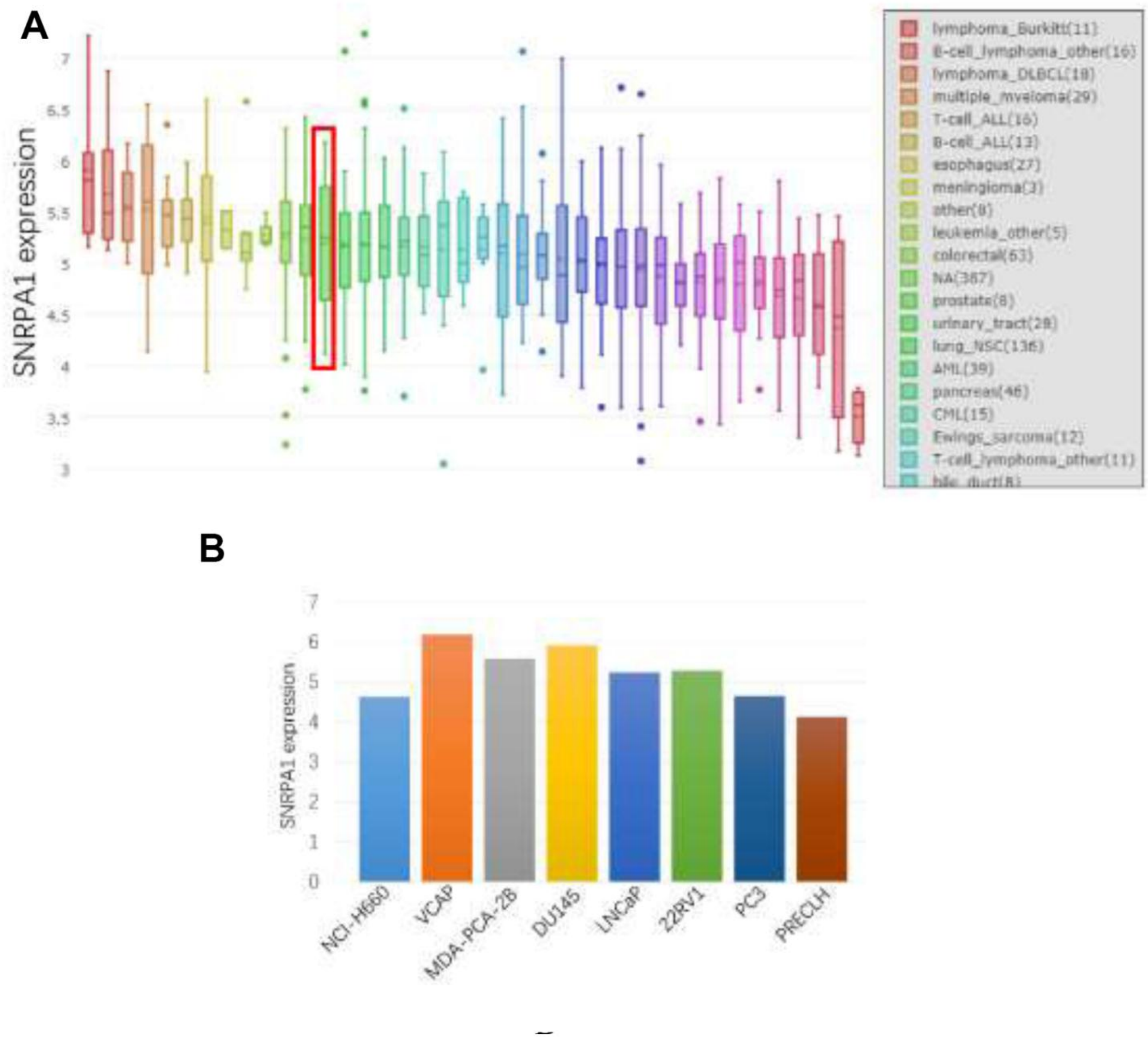

Supplementary Figure 1. The expression profile of SNRPA1 in different tumors (**A**) and PCa cell lines (**B**) in CCLE. PCa = Prostate cancer; CCLE = Cancer Cell Line Encyclopedia.
