# Supplementary material for: Identification of RNA-binding protein SNRPA1 for prognosis in prostate cancer
Source: Aging (Albany NY). 2021 Jan 15;13(2):2895–911. doi: 10.18632/aging.202387 (PMC7880319; doi:10.18632/aging.202387)
Supplement: Supplementary Table 1 [file aging-13-202387-s002.pdf]

SUPPLEMENTARY TABLES

Supplementary Table 1. The results of univariate and multivariate Cox regression analyses between RBPs and DFS.

	Univariate analysis		Multivariate analysis	
**RBP**	**HR (95%CI)**	**p value**	**HR (95%CI)**	**p value**
TRMU	1.113(1.001-1.239)	0.049		
DDX39B	1.072(1.034-1.112)	< 0.001	1.067(1.028-1.107)	< 0.001
SNRPF	1.046(1.009-1.084)	0.015		
SNRPA1	1.187(1.079-1.310)	< 0.001	1.158(1.041-1.287)	0.006
SNRNP70	1.013(1.005-1.021)	0.001		
POLR2H	1.050(1.014-1.087)	0.007		
ESRP2	0.958(0.922-0.994)	0.024	0.962(0.927-0.999)	0.042

RBPs: RNA-binding proteins; HR: hazard ratio; CI: confidential interval; DFS: disease-free survival.
